# Molecular Interactions between Prions as Seeds and Recombinant Prion Proteins as Substrates Resemble the Biological Interspecies Barrier *In Vitro*

**DOI:** 10.1371/journal.pone.0014283

**Published:** 2010-12-09

**Authors:** Giannantonio Panza, Lars Luers, Jan Stöhr, Luitgard Nagel-Steger, Jürgen Weiβ, Detlev Riesner, Dieter Willbold, Eva Birkmann

**Affiliations:** 1 Institut für Physikalische Biologie, Heinrich-Heine-Universität Düsseldorf, Düsseldorf, Germany; 2 Institut für klinische Biochemie und Pathobiochemie, Deutsches Diabetes-Zentrum, Düsseldorf, Germany; 3 Institut für Strukturbiologie und Biophysik 3, Forschungszentrum Jülich, Jülich, Germany; University Paris 7, France

## Abstract

Prion diseases like Creutzfeldt-Jakob disease in humans, Scrapie in sheep or bovine spongiform encephalopathy are fatal neurodegenerative diseases, which can be of sporadic, genetic, or infectious origin. Prion diseases are transmissible between different species, however, with a variable species barrier. The key event of prion amplification is the conversion of the cellular isoform of the prion protein (PrP^C^) into the pathogenic isoform (PrP^Sc^). We developed a sodiumdodecylsulfate-based PrP conversion system that induces amyloid fibril formation from soluble α-helical structured recombinant PrP (recPrP). This approach was extended applying pre-purified PrP^Sc^ as seeds which accelerate fibrillization of recPrP. In the present study we investigated the interspecies coherence of prion disease. Therefore we used PrP^Sc^ from different species like Syrian hamster, cattle, mouse and sheep and seeded fibrillization of recPrP from the same or other species to mimic *in vitro* the natural species barrier. We could show that the *in vitro* system of seeded fibrillization is in accordance with what is known from the naturally occurring species barriers.

## Introduction

Prion diseases are fatal progressive neurodegenerative diseases of spontaneous, genetic, or infectious origin. The conversion of the host encoded prion protein (PrP^C^) into the disease causing isoform PrP^Sc^ is the key molecular event in prion disease. The common hypothesis is, that the amplification of PrP^Sc^ is achieved by the conversion of the α-helical dominated cellular isoform PrP^C^ into β-sheet rich and insoluble PrP^Sc^ while PrP^Sc^ acts as template and catalyst for PrP^C^ conversion [1]. The pool of PrP^C^ is replenished by the cellular synthesis of PrP^C^. Some mechanistic models have been suggested, including the heterodimer model [2], the cooperative model [3], and the model of seeded polymerization [4]. Most experimental data support the seeded polymerization. Prion diseases are not only transmissible within one species, but in some cases also between different species [5]. This so called “interspecies transmission” is of special interest since the origin of bovine spongiform encephalopathy is suspected to be caused by feeding meat and bone meal from Scrapie infected sheep to cattle, i.e. an interspecies transmission might have lead to the BSE-epidemic in the UK [6]. In an experimental approach it was indeed shown that cattle are susceptible to infection by sheep Scrapie isolates [7]. On the other hand it is known that interspecies transmission can fail e.g. Syrian hamster Scrapie is not transmissible to mice [8]. In that case the interspecies barrier would be too high for experimental observation. The molecular mechanism of prion disease transmissibility between different species is still not understood.

A molecular model for the infectious process of prions is the conversion of PrP^C^ to PrP^Sc^ induced by the invading PrP^Sc^. As mentioned above, the widely accepted mechanistic model is that of PrP^Sc^ acting as seed for polymerization of PrP^C^ [9], [4], [10]. To study the mechanism of spontaneous and seed-depended fibrillization of recPrP different *in vitro* conversion assays were introduced, which lead to the formation of amyloid fibrils [11], [12], [13]. The *in vitro* conversion into amyloid led to the first generation of synthetic prions utilizing only murine recPrP [14], i.e. without PrP^Sc^ as seed, which would represent a model for the sporadic case of prion diseases.

In the present study we use the SDS PrP conversion system to simulate intra- and interspecies transmission *in vitro*. It is a minimal system in the sense, that only recPrP as substrate, buffer, and purified PrP^Sc^ as seed, but no cellular extract is needed. We extend the spontaneous and seed depended conversion, established for recombinant Syrian hamster PrP [11] and bovine recPrP [15], to recombinant ovine and murine PrP. It has been shown earlier that well balanced concentrations of sodium chloride and SDS have to be chosen for every substrate PrP in order to guarantee a suitable extent of partial denaturation, which is a prerequisite for fibrillization, spontaneous as well as seeded fibrillzation.

To monitor amyloid formation of recPrP we used the amyloid specific marker Thioflavin T (ThT). We are able to mimic the species barrier *in vitro* in complete accordance with the species barrier as found with the *in vitro* infection of prions.

## Results

In our previous studies we analyzed spontaneous and PrP^Sc^-seeded fibril formation of recombinant PrP (recPrP). Buffer conditions were established which consist of well selected SDS and NaCl concentrations, so that recPrP forms spontaneously amyloid fibrils within weeks, but the fibril formation was accelerated by seeding with PrP^Sc^ to hours or days. It is important to note, that the optimal buffer conditions had to be selected for the species of recPrP during spontaneous fibril formation, and these conditions were used for seeded fibril formation. It was discussed earlier that the buffer conditions allow a particular extent of partial denaturation of recPrP in a well characterized pre-amyloid state. Spontaneous and seeded fibril formation of recPrP was analyzed with bovine and hamster recPrP as substrate and the homologous NaPTA-precipitated PrP^Sc^ as seeds [11], [15]. In the present study, we established conditions for spontaneous and seeded fibril formation of recPrP from sheep (aminoacids: 25–233) and mouse (aminoacids 89–231). The aim of this study was to combine recPrP-substrates and PrP^Sc^-seeds of different species to investigate if the fibril formation *in vitro* does resemble the well known phenomenon of species barrier for transmissibility. In our system the species barrier phenomena are studied on the level of the molecular interaction of PrP and PrP^Sc^.

### Spontaneous fibril formation of recombinant ovine and murine prion protein

**Figure 1:**
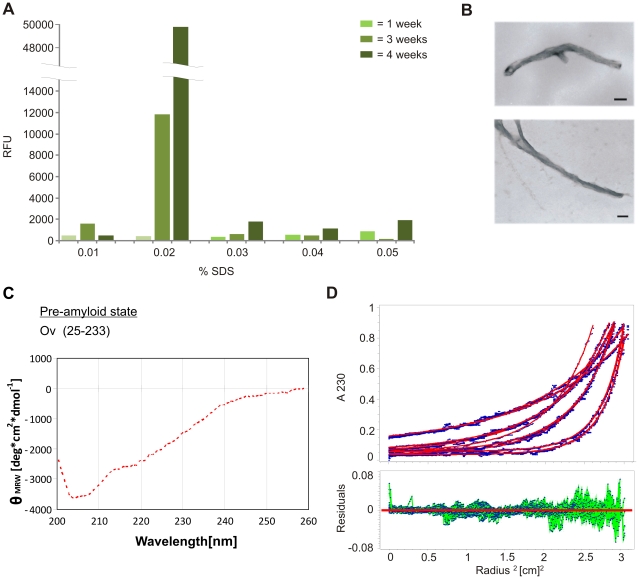
Characterization of spontaneous OvPrP(25–233) fibril formation within the *in vitro* conversion system. **A**: Dependence of OvPrP(25–233) amyloid formation on SDS-concentration. OvPrP(25–233) was incubated in 10 mM NaPi pH 7.4, 250 mM NaCl and 0.01–0.05% SDS. The amount of fibril formation was measured by ThT-fluorescence. Thioflavin T was added to a final concentration of 5 µM to 10 ng/µl OvPrP(25–233). **B**: Electron micrographs show the typical structure of amyloid fibrils after 7 days of incubation of OvPrP(25–233) in 10 mM NaPi pH 7.4, 250 mM NaCl 0.02% SDS (bar = 20 nm). **C**: Secondary structure analysis of the pre-amyloid state. CD-spectra were measured directly after adapting the SDS conditions, with a final concentration of 150 ng/µl OvPrP(25–233) in 10 mMNaPi pH 7.4 and 250 mMNaCl. **D**: Sedimentation equilibrium centrifugation of OvPrP(25–233) after 7 days of incubation. (*left*) Experimental data overlaid by the fitted curves (*right*) residuals.

To determine optimal buffer conditions in which ovine PrP (OvPrP(25–233)) forms amyloid fibrils, we analyzed OvPrP(25–233) fibril formation in 10 mM NaPi, 250 mM NaCl by varying the SDS-concentration from 0.01 to 0.05%. Fibril formation was followed in the Thioflavin T assay. Incubation was carried out at 37°C under constant agitation for 4 weeks and fibrils could be observed at 0.02% SDS after three to four weeks (fig. 1A). The fibrillar character of OvPrP(25–233) was verified by electron microscopy (fig. 1B). At higher or lower SDS-concentrations amyloid specific fluorescence increase could not be observed. Therefore in all the following experiments with OvPrP(25–233), the buffer was NaPi pH 7.2, 250 mM NaCl and 0.02% SDS. As mentioned above amyloid formation depends upon of a specific pre-amyloid state of recPrP as long as it is soluble [11], [15]. Therefore we analyzed also the pre-amyloid state of OvPrP(25–233). The secondary structure, revealed with circular dichroism, showed in some contrast to formerly analyzed initial-states a higher random-coil amount (fig. 1C). A comparison of the secondary structure analysis of different species are shown in Text S1. In line with previous studies [11] the analytical ultracentrifucation data revealed an equilibrium of monomeric (33%) and dimeric (67%) OvPrP(25–233) (fig. 1D, Text S1).

Buffer conditions for fibril formation of murine recPrP (MuPrP(89–231)) were analyzed in the same way. It was found that a wider SDS-range was suitable for fibril formation, with an optimum at 0.04% SDS in NaPi pH 7.4, 250 mM (data not shown).

### Intraspecies PrP^Sc^-seeded amyloid formation

**Figure 2:**
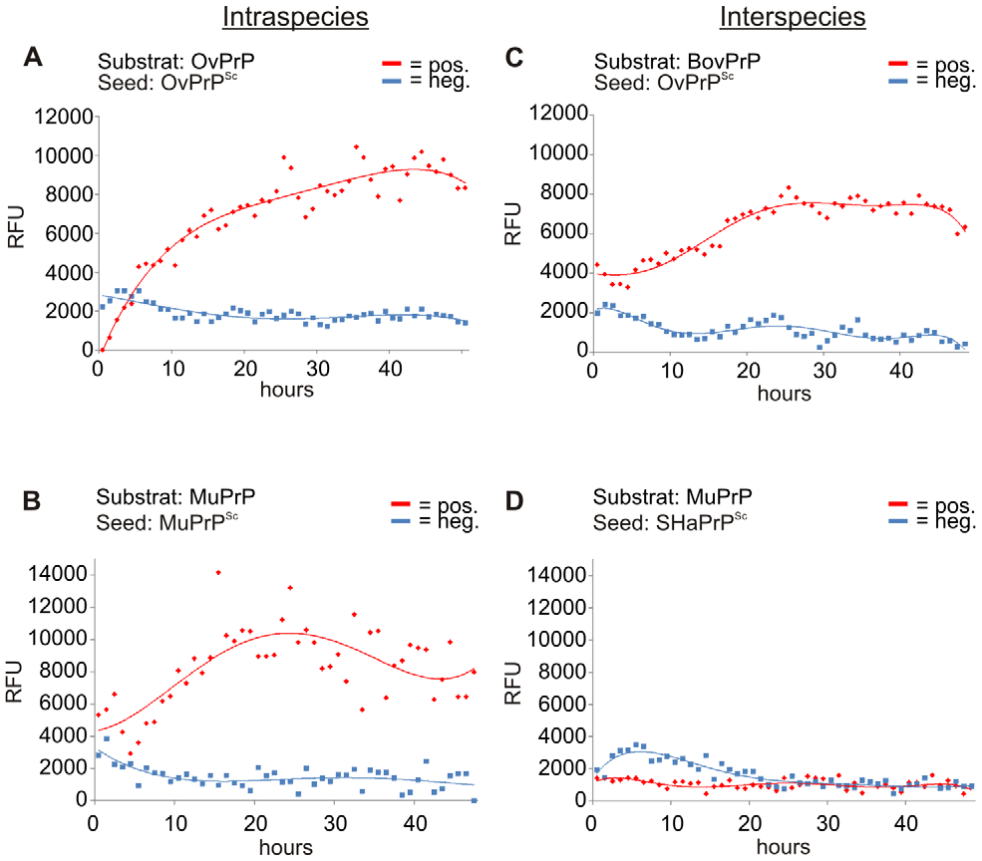
Kinetics of intra- and interspecies seeded recPrP amyloid formation. Amyloid formation of recPrP was monitored using ThT-fluorescence assay in 10 mM NaPi (pH 7.4) with 250 mM NaCl (at 37°C). *In vitro* intraspecies transmission: (**A**) Amyloid formation of 1.8 mM (40 ng/µl) OvPrP(25–233)-substrate seeded with OvPrP^Sc^ in 0.02% SDS (red line), (**B**) of 1.8 mM (30 ng/µl) MuPrP(89–231)-substrate seeded with MuPrP^Sc^ in 0.04% SDS (red line). *In vitro* interspecies transmission: (**C**) Amyloid formation of BovPrP(25–241)-substrate seeded with OvPrP^Sc^ in 0.02% SDS (red line). *In vitro* interspecies barrier: (**D**) In presence of SHaPrP^Sc^ no MuPrP(89–231) amyloid formation occurs (red line). NaPTA-precipitate of corresponding same treated brain tissue from healthy animals: blue line.

In previous studies we showed that NaPTA-precipitated PrP^Sc^ from brain homogenate works very well as seed and could drastically accelerate fibril formation. As a control, NaPTA-precipitate from brain homogenate of uninfected animals did not show any acceleration effect. The presence of the N-terminal sequence (aminoacids 23–89) had no influence on the seeding effect (Text S1). For the ovine system, NaPTA-precipitated PrP^Sc^ from brain tissue of Scrapie-infected sheeps (OvPrP^Sc^) was used as seed and accelerated fibril formation was observed based on the increase of ThT-fluorescence within 10 to 20 hours (fig. 2A). The approach was carried out with 40 ng/µl rec OvPrP(25–233) (1.8 µM) in conversion buffer and 0.02% SDS.

We also adopted the seed dependent fibril formation for MuPrP. NaPTA-precipitated PrP^Sc^ from mouse brain accelerated the process down to 10 to 20 hours (fig. 2B) while the spontaneous formation of amyloids takes at least 7 days (data not shown). As a control NaPTA-precipitate of non-infected mouse brain tissue did not lead to any formation of amyloidogenic fibrils in the time range of the experiment.

### In vitro species barrier

The aim of this study was to analyze the species barrier of prion infection *in vitro*, i.e. with PrP^Sc^-seeds and otherwise purified components only. From natural and experimental transmission data it is known that prion diseases are transmissible in some cases from one species to another and in other cases not. This phenomenon is commonly known as species barrier. PrP^Sc^ from brain homogenate of one species was taken as seeds and recPrP of another species as template. Transmission was simulated by fibrillzation in our *in vitro* conversion assay with the exact concentrations of that particular species.

As an example of interspecies transmission of ovine Scrapie to cattle as recipient [7] is shown in fig. 2C using OvPrP^Sc^ as seed and BovPrP(25–241) as substrate. Accelerated fibril formation of BovPrP by OvPrP^Sc^-seed is clearly observed, very similar to the intraspecies seeded fibrillization in sheep (fig. 2A).

In another experiment, murine recPrP was seeded with prions from Scrapie infected hamster. In contrast to the intraspecies seeded fibrillization of MuPrP(89–231) (fig. 2B), no increase of the ThT-fluorescence was measured within a time period of 48 hours (fig. 2D). This shows, that SHaPrP^Sc^ does not act as seed for accelerated fibril formation of MuPrP(89–231). This corresponds to the well known species barrier between Syrian hamster Scrapie and mouse as recipient [8]. In all seeded fibrillization experiments, i.e. intraspecies as well as interspecies combinations, NaPTA- precipitate of brain tissue from non-infected animals served as controls and did not lead to an increased fluorescence read out within the timescale of the experiments (fig. 2 blue lines).

**Figure 3:**
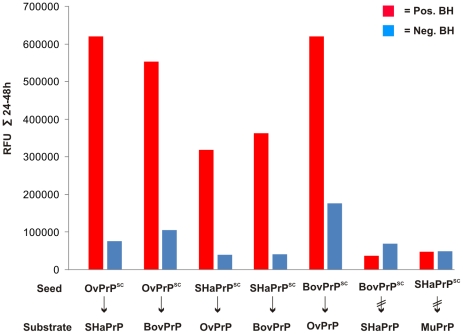
Comparison of different interspecies recPrP seeding. Amyloid formation in the seeding assay was monitored using Thioflavin T. The fluorescence signals were recorded every 30 min at 37°C at recPrP amyloid forming conditions. Fluorescence intensities were summarized from 24–48 h (saturation phase). (Left to right) Interspecies transmission: OvPrP^Sc^ in SHaPrP(90–231); OvPrP^Sc^ in BovPrP(25–241); SHaPrP^Sc^ in OvPrP(25–233); SHaPrP^Sc^ in BovPrP(25–241); BovPrP^Sc^ in OvPrP(25–233). Interspecies barrier: BovPrP^Sc^ in SHaPrP(90–231); SHaPrP^Sc^ in MuPrP(89–231). As control parallel an approach was made with the corresponding intraspecies transmission (data not shown).

Figure 3 gives an overview of the fluorescence readouts of various seed - substrate combinations. The ThT-fluorescence intensities in the time period of 24 to 48 hours of incubation and after seeding were summarized. Interspecies transmission is obvious for five different combinations of PrP^Sc^-seeds and recPrP substrate: OvPrP^Sc^ to SHaPrP(90–231), OvPrP^Sc^ to BovPrP(25–241), SHaPrP^Sc^ to OvPrP(25–233), SHaPrP^Sc^ to BovPrP(25–241) and BovPrP^Sc^ to OvPrP(25–233). In our system, approach two well known *in vivo* interspecies barriers [8] [16] could be simulated with our *in vitro*, conversion system namely the species barrier of BSE to Syrian Hamster as well as of Syrian Hamster Scrapie to mouse. In summary, all of our *in vitro* intra- and interspecies transmission results resemble exactly the *in vivo* situation (table 1).

**Table 1:** *In vivo* vs. *In vitro* intraspecies and interspecies transmission and species barrier.

in vivo				
	BovPrP	OvPrP	SHaPrP	MuPrP
BovPrP^Sc^	**+**	**+**	**−**	**+**
OvPrP^Sc^	**+**	**+**	**+**	**+**
SHaPrP^Sc^	**(+)**	**(+)**	**+**	**−**
MuPrP^Sc^	**(+)**	**(+)**	**+**	**+**

in vitro				
	BovPrP	OvPrP	SHaPrP	MuPrP
BovPrP^Sc^	**+**	**+**	**−**	n.d.
OvPrP^Sc^	**+**	**+**	**+**	n.d.
SHaPrP^Sc^	**+**	**+**	**+**	**−**
MuPrP^Sc^	n.d.	n.d.	n.d.	**+**

**+**  =  Intraspecies transmission; +  =  Interspecies transmission; −  =  Interspecies barrier; (+)  =  transmission studies in transgenic mice; n.d. not determined.

## Discussion

Conversion of the cellular prion protein (PrP^C^) to PrP^Sc^ is the key event in prion infection [2]. Although many studies with recPrP and with different conversion systems have been carried out the molecular mechanism is still not well understood [17] [18], [19]. These studies are based mainly on the simulation of the structural properties of PrP^Sc^ like β-sheet content, PK-resistance or morphology of amyloid fibrils. However these systems have not generated infectious PrP or only very low titers of infectivity were generated spontaneously [14]. Recently, the group of S.B. Prusiner and colleagues could show that subtle variations in the structure of *in vitro* generated fibrils give rise to a variety of infectious preparation with distinct strain properties [20]. In contrast to spontaneous fibrillization seed-dependent assays like protein misfolding cyclic amplification (PMCA) [21] and quaking-induced conversion (Quic) [22] were established and are in very good agreement with the infectious etiology of prion diseases. Both assays are carried out in cellular extracts from uninfected animals or cells, which cannot exclude the involvement of cellular compounds in the conversion reaction.

Our group established a SDS-based conversion assay that works without cellular extracts, using solely purified compounds, like phosphate buffer, recPrP as substrate and prepurified PrP^Sc^ as seed [11]. The use of partially denaturing conditions in conversion of recombinant PrP into amyloid with physiochemical properties reminiscent of PrP^Sc^ seems to be a general concept in *in vitro* conversion studies of PrP and were studied in greater detail [18], [22]. Beside those classical protein denaturants other compounds have been identified (e.g. glycosaminclycans and oligonucleotides) to promote PrP conversion *in vitro* but their structural influence on PrP remains unknown [23], [24].

PMCA and Quic were mainly developed for diagnostic purposes, our system with the well defined components was developed to describe quantitatively the prion propagation mechanism using hamster PrP. This SDS-based conversion assay was applied to additional species, in order to test if our conversion system can be used with prion proteins of different species and if the pre-amyloid state described with hamster PrP represent a general mechanism for amyloid formation in our in vitro conversion system. Furthermore, by combining seed and substrate from different species we have the opportunity to simulate the phenomena of species barriers for the first time on the level of a direct molecular interaction of prion seed and recPrP substrate without the influence of any other cellular component. In the presented study we were able to show that amyloid fibrils of recPrP can be formed within the SDS-based conversion system for all species investigated (cattle, sheep, mouse, hamster). Only the SDS concentration had to be adapted. These results and the properties of the intermediate state will be discussed on a more detailed level later. The phenomenon of species barrier for prion transmission was successfully modeled as seed dependent *in vitro* fibrillization. Five interspecies transmissions have been observed (fig. 3). Not very surprisingly, the amyloid formation of interspecies transmission seems to be slower as compared to *intra*species transmission (fig. 2). Two interspecies transmissions were reported to have failed (SHaPrP^Sc^ to MuPrP(89–231) and BovPrP^Sc^ to SHaPrP(90–231)). Consequently, also a strict species barrier was found in the *in vitro* simulation (fig. 3). For murine substrate, only one positive transmission (MuPrP^Sc^ to MuPrP(89–231)) and one negative (SHaPrP^Sc^ to MuPrP(89–231)) could be performed for reasons of shortage of mouse prions in our lab. As a main result of our work we found that the *in vitro* simulation of the species barrier is in complete agreement with the experimental data from *in vivo* transmission studies (Tab. 1). Similar results on interspecies transmission and species barrier were reported by studies with PMCA [25] and by Cashman and co-workers [26], whereby both systems use cellular extracts. Because our conversion system does not include cellular extracts we conclude that the species barrier is encoded within the direct interaction of PrP^Sc^ and PrP^C^ [27]. Interspecies transmission as well as species barrier is well resembled in our system. Cellular factors might be beneficial for the conversion reaction, but only by enforcing the pre-existing interaction.

The interpretation of our results on the molecular level shows the importance of partial denaturation of recPrP as substrate as described by Stöhr *et al.* [11]. PrP in this intermediate or pre-amyloid state is soluble for weeks, suggesting that it is in a state of low free energy. Since it is present in a monomer-dimer-equilibrium one can argue that the partially denaturated PrP is prone to intermolecular interactions possibly also with PrP^Sc^. However in our conversion conditions PrP is in the state of lowest free energy if it is refolded for attachment to the fibrillar seed. We assume that this state is not present in measurable amounts in solution but only attached to the seed. The degree of denaturation in the intermediate state is critical; for different recPrP sequences, i.e. for different species, different SDS concentrations are needed. More than the optimal SDS concentration would lead to a more unfolded PrP^C^ whereas less SDS leads to a more refolded PrP^C^ state as compared to the optimal intermediate state. In both cases the conversion of PrP to fibrillar PrP would be too slow to be observable. For an interspecies transmission a partially unfolded state of the substrate PrP is required, but ΔG for substrate (PrP refolded in the complex) with the seed from the other species in the same way as for intraspecies transmission has to be sufficiently low, i.e. lower than in the intermediate state, to guarantee the transition. In summary not the intermediate state of substrate PrP is critical for transition but its potency to refold into a conformation well adapted to the PrP^Sc^-seed.

In future experiments we will extend our experimental approach to other species like human CJD and cervid CWD. Due to the high occurrence of CWD in Northern America, the combination of CWD-seed and human PrP as substrate would be of particular interest.

## Materials and Methods

### Recombinat Prion Proteins

The recombinant prion protein (recPrP) was prepared and purified as described previously [28], [29]. The recPrP with the amino acid sequence of Syrian Hamster (90–231) (SHaPrP) PrP as well as recPrP with the amino acid sequence of cattle (25–241) (BovPrP) PrP was used in our studies before [15]. We adopted the purification protocols to full length recombinant ovine PrP (25–233) (OvPrP). Recombinant murine PrP (89–231) (MuPrP) was acquired from Allprion (Schlieren, Switzerland).

### NaPTA precipitation of PrP^Sc^

PrP^Sc^ from brain tissue of different species was purified by NaPTA (Sodium phosphotungstate dibasic hydrate) precipitation [30], [31] Additionally to Syrian hamster and bovine PrP^Sc^ (SHaPrP^Sc^ and BovPrP^Sc^) [11], [15] we adopt the purification protocol to ovine PrP^Sc^ (OvPrP^Sc^) and to Murine PrP^Sc^ (MuPrP^Sc^). The resulting pellet was resuspended in 10 mM NaPi by brief sonification (Sonificator, Labsonic U, Braun Dissel, Melsungen).

### Circular dichroism spectroscopy

Circular dichroism (CD) spectra were recorded with a J-715 spectropolarimeter (Jasco, Easton, MD, USA) in a 0.1 cm quartz cuvette at room temperature. The scanning speed was 50 nm/min with resolution of 1 nm. For each sample 10 spectra were accumulated between 195 and 260 nm. The protein concentration was 150 ng/µl. Background spectra of buffer samples were subtracted from the respective protein spectra.

### Analytical Ultracentrifugation

Sedimentation-diffusion equilibrium experiments were performed, as described in ref [11], in a Beckman Optima XL-A analytical ultracentrifuge (Beckman Coulter) applying standard 12-mm double-sector cells at 20°C. The data were analyzed by using the Global Fit procedure, which is implemented in the UltraScan II software package (Version 5.0 for UNIX) of B. Demeler (University of Texas Health Science Center, San Antonio, TX).

### Electron microscopy, negative stain

A droplet of 5–10 µl containing the recPrP was placed on glow discharged grid and left to adsorb for 2 minutes. After adsorption to the grid surface the sample was washed briefly (50 µl of: 0.1 and 0.01 M NH_4_ acetate) and stained with 2% ammoniummolybdate (50 µl). The samples were analyzed with a Zeiss EM910 microscope at 80 kV.

### Thioflavin T-Assay

Fluorescence emission spectra of Thioflavin T (ThT) were measured at a concentration of 5 µM ThT and 10 ng/µl recPrP in 150 µl 10 mM NaPi pH 7.4. The emission spectra were recorded from 460 nm to 630 nm with a fixed excitation wavelength of 455 nm, average of λ_em_ 495 to 505 is shown for a time point. Fibrillization kinetics were followed in 96 well plates according to Stöhr *et al.* [11]. All measurements were performed in a Tecan saphire plate reader (Tecan Group, Maennedorf, Switzerland). The chosen regression line is polynomial fitted to original data points.

### Spontaneous and seeded amyloid formation of recPrP

Spontaneous and seeded amyloid fibril formation of recPrP of the different species was monitored by ThT-assay as described above. The buffer conditions especially the SDS-concentrations were adjusted according to the amyloid forming conditions of the spontaneous case for each species. The results are displayed as sum of the average fluorescence intensities of 495 to 505 nm in the saturation phase (24 h to 48 h in 30 min interval) of the curve. To determine the specificity of the seeding effect in interspecies transmission or interspecies barrier NaPTA-precipitated PrP^Sc^ from brain tissue of infected and non-infected animals was compared.

## Supporting Information

Text S1 Including supporting figures and tables.
(0.13 MB DOC)
