# Supplementary material for: Molecular Interactions between Prions as Seeds and Recombinant Prion Proteins as Substrates Resemble the Biological Interspecies Barrier In Vitro
Source: PLoS One. 2010 Dec 9;5(12):e14283. doi: 10.1371/journal.pone.0014283 (PMC3000319; doi:10.1371/journal.pone.0014283)
Supplement: Text S1 — Including supporting figures and tables. (0.13 MB DOC) [file pone.0014283.s001.doc]

***S1 supplementary material*:**


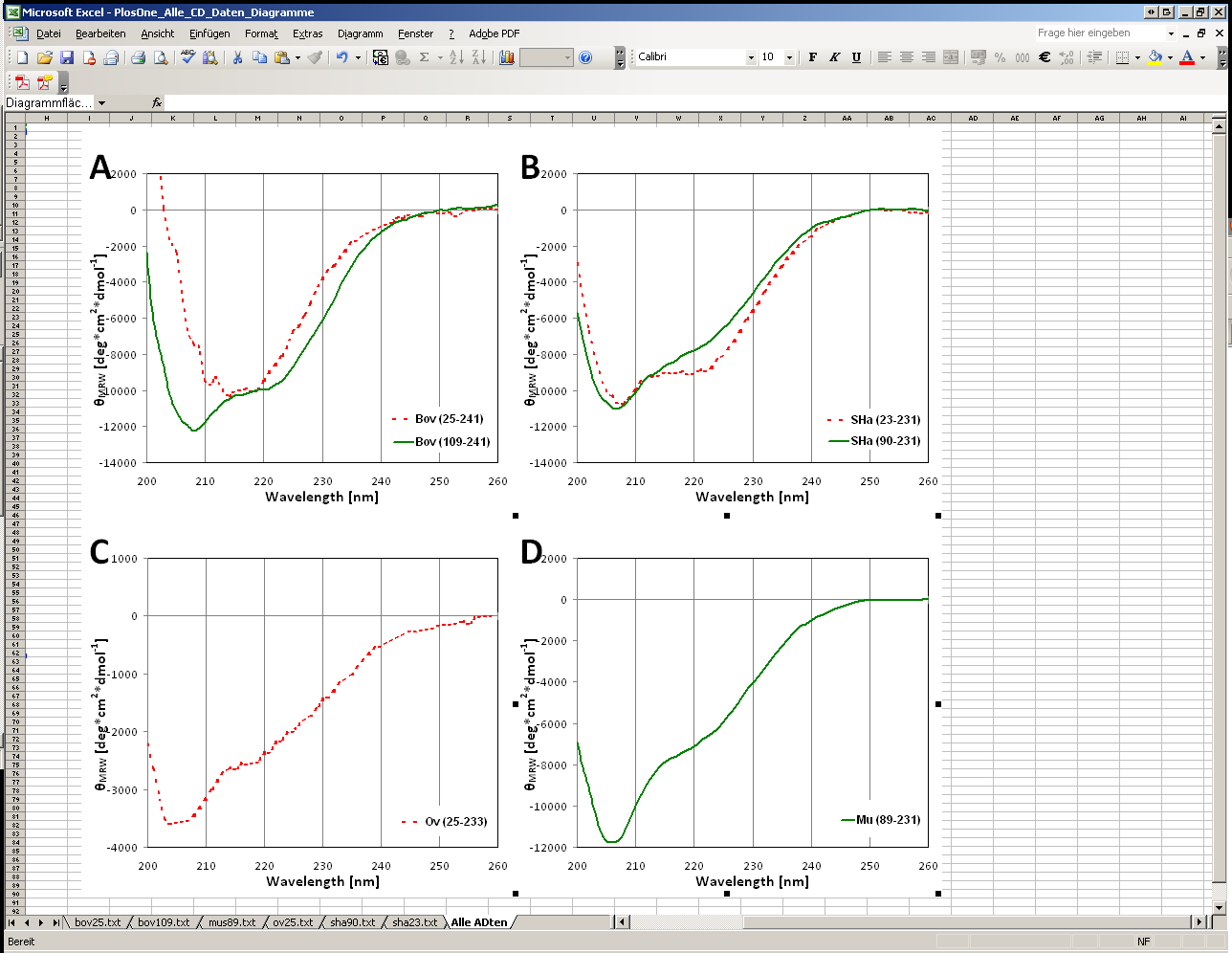


**Fig. S1-1**

Secondary structure analysis of the pre-amyloid state: CD-spectra were measured directly after adapting the SDS conditions, with a final concentration of 150 ng/µl recPrP in 10 mMNaPi pH 7.4 and 250 mMNaCl.

|  | % monomer | % dimer |
| --- | --- | --- |
| hamster | 33 | 67 |
| bovine | 33 | 67 |
| ovine | 33 | 67 |

Tab. S1-2

Ratio of monomeric and dimeric recPrP analyzed by sedimentation equilibrium centrifugation corresponding to Fig 1 D.

The corresponding sedimentation equilibrium centrifugation analysis are shown: hamster [11], bovine [15], ovine (see fig. 1)

Fig. S1-3:

Kinetics of SHaPrP^Sc^ seeded SHaPrP amyloid formation - comparison fullength and truncated sequences of substrate.

Amyloid formation of SHaPrP (red: fullength sequence aa23-231; blue: truncated sequence aa90-231), was monitored using ThT-fluorescence assay in 10 mM NaPi (pH 7.4) with 250 mM NaCl, 0.03% SDS (at 37°C).
